# Difference between spinecor brace and rigid brace during treatment

**DOI:** 10.1186/1748-7161-8-S1-P8

**Published:** 2013-06-03

**Authors:** Ö Ersen, B Bilekli, S Bilgic, E Oguz, A Sehirlioglu

**Affiliations:** 1 Gulhane Military Medical Academy, Ankara, Turkey

## Background

Brace treatment in idiopathic scoliosis is the only efficacious method of non operative treatment. The effectiveness of dynamic SpineCor brace with corrective movement principle has been shown, but differences between rigid braces and SpineCor brace is still unclear.

## Aim

The aim of this study is to evaluate differences between rigid brace and SpineCor brace in terms of curve progression, spinal height increase, and SRS-22 questionnaire during treatment.

## Methods

A total of 76 consecutive adolescent idiopathic scoliosis patients who were treated with brace were included in this study. 45 patients were treated with SpineCor brace, 31 patients were treated with rigid braces. After detailing braces and their costs, choice was made by patients’ family. Patient’s height, T1-Coccyx distance, gibbosity, and Cobb angles were documented at the beginning of the treatment and last control. At last visit SRS-22 questionnaire applied to the patients to evaluate clinical effect of braces.

## Results

Average age of SpineCor group was 12.8±1.5 and average follow up period was 25±10.6 months. In rigid brace, the group average age was 12.2±1.3 and average follow up period was 23±6.7months. There were no differences between groups according to age, gender, height, T1-Cx distance, Cobb angles, gibbosity before brace treatment initiated. In both groups, height and T1-Cx distance increased and there were no difference. Cobb angle decreased 1.5° in SpineCor group and increased 1.1 ° in rigid brace group (p=0,137). Gibbosity decreased 0.6° in SpineCor group and increased 0.3° in rigid brace group (p=0,086). According to SRS-22 questionnaire, SpineCor brace patients’ pain, self image and activity/function scores were statistically better than rigid brace patients’ scores, while mental health and satisfaction from treatment scores were similar.

## Conclusions

Although SpineCor brace and rigid braces have similar effects on curve correction, height and spinal height, the real benefits of SpineCor brace is less pain, less anxiety about self image and more activity, and function.
